# Pacing in World-Class Age Group Swimmers in 200 and 400 m Individual Medley

**DOI:** 10.3389/fphys.2020.629738

**Published:** 2021-01-20

**Authors:** Cathia Moser, Caio Victor Sousa, Rafael Reis Olher, Lee Hill, Pantelis Theodoros Nikolaidis, Thomas Rosemann, Beat Knechtle

**Affiliations:** ^1^ Balgrist University Hospital, Zurich, Switzerland; ^2^ Bouvé College of Health Sciences, Northeastern University, Boston, MA, United States; ^3^ University Center of Central Plateau Apparecido dos Santos, Brasília, Brazil; ^4^ Division of Gastroenterology and Nutrition, Department of Pediatrics, McMaster University, Hamilton, ON, Canada; ^5^ Exercise Physiology Laboratory, Nikaia, Greece; ^6^ School of Health and Caring Sciences, University of West Attica, Athens, Greece; ^7^ Institute of Primary Care, University Hospital Zurich, Zurich, Switzerland; ^8^ Medbase St. Gallen Am Vadianplatz, St. Gallen, Switzerland

**Keywords:** world class level, pacing, freestyle, backstroke, butterfly, breaststroke

## Abstract

The present research investigated pacing for world-class age group swimmers competing in individual medley in 200 m and 400 m. Data on 3,242 unique finishers (1,475 women and 1,767 men) competing in four Master World Championships [XV FINA WMC held in Montreal (Canada) in 2014, the XVI FINA WMC held in Kazan (RUS) in 2015, the FINA WMC held in Budapest (HUN) in 2017, and the XVIII FINA WMC held in Gwangju (KOR] in 2019) were analyzed. Men were faster than women among all age groups in both 200 and 400 m. Additionally, differences were found between almost all adjacent age groups, with the exception (*p* > 0.05) of age groups 25–29 to 30–34, 35–39 to 40–44 years in 200 m races and 25–29 to 30–34, 30–34 to 35–39, 35–39 to 40–44, and 45–49 to 50–54 years in 400 m races. Men showed a higher pacing variation in 200 m among all male age groups and all female age groups up to 69 years. Pace-variation pairwise comparisons between men and women showed no consistencies throughout age groups, with the exception of a higher variation in men in age groups ≥55-year-old. Men were faster for all splits and strokes in both 200 and 400 m, and significant changes were identified for each split and stroke for both men and women in both 200 and 400 m. Front crawl (freestyle, 4th split) was the fastest butterfly (1st split), backstroke (2nd split), and breaststroke (3rd split). In summary, men were faster than women for all age groups in both 200 and 400 m. Men showed a higher pacing variation in 200 m in all age groups, where women had a higher variation in age groups up to 69 years. The fastest stroke for the final spurt was front crawl, followed by butterfly, backstroke, and breaststroke. Based on these findings, coaches should advise their master athletes to focus on the final spurt in both 200 and 400 m individual medley for a fast final race time.

## Introduction

Participation in master-level swimming competitions has remarkably increased over the last 30 years (Ransdell et al., 2009; Mejias et al., 2014; Ferreira et al., 2016) and has seen an extraordinary growth in popularity since the first Fédération Internationale de Natation (FINA) World Masters Championship (WMC; Rubin, 2010) held in Tokyo in 1986. Swimming is one of the most popular sports among master athletes, attracting both casual and elite level swimmers (Ferreira et al., 2016). However, in recent years, there has been a noticeable shift from a recreational to a more elite performance while competing in WMC (Mejias et al., 2014).

The volume of pacing research has continuously grown over the last few years and is mainly dominated by endurance sports, such as running, cycling, and triathlon (McGibbon et al., 2018). However, little is known about pacing strategies in master swimmers (Nikolaidis and Knechtle, 2017; Moser et al., 2020). In contrast, most of the studies on master swimmers focused more on biomechanical and physiological determinants of performance and also how these determinants varied by age (Reaburn and Dascombe, 2008; Tanaka and Seals, 2008). Generally, pacing strategies are described as how an athlete distributes work and energy throughout an exercise task (Abbiss and Laursen, 2008). Accordingly, an athlete can select the most appropriate pacing strategy, which can include negative, all-out, positive, even, parabolic, and variable pacing (Abbiss and Laursen, 2008). Pacing in swimming is determined by plotting split times or velocity over each lap of the event (McGibbon et al., 2018).

In more recent years, research around swimming pacing has been mainly focused on freestyle swimming (Robertson et al., 2009; Mauger et al., 2012; Nikolaidis and Knechtle, 2017; Moser et al., 2020). Further, it has been noted in long-distance swimming events that the speed profile of 800 and 1,500 m international-level freestyle swimmers was generally parabolic or U-shaped (Damasceno et al., 2013). In this instance, the swimmers completed the first segment in the fastest time (assisted by the dive start), followed by a marked decrease in speed during the middle portion and then an increased speed toward the final segment of the race (Abbiss and Laursen, 2008). Further, elite 400 m freestyle swimmers have been shown to adopt a similar profile and used fast-start-even and parabolic pacing strategies (Mauger et al., 2012).

More recently, research on elite master level age group swimmers started to focus on longer swimming distance events, such as the 400 m freestyle (Lipinska and Hopkins, 2020) and the 800 m freestyle (Lipińska et al., 2016). Although previous studies have investigated performance and age progression within elite age group swimmers, little is known about age group swimmers competing at international level in the individual medley events (Saavedra et al., 2012; McGibbon et al., 2018). The individual medley is a unique event combining all four different swimming styles in the order of butterfly (1st split), backstroke (2nd split), breaststroke (3rd split), and freestyle (front crawl, 4th split) in one race (Rubin, 2010). This order might, indeed, affect pacing of the entire race (Dormehl et al., 2016). Accordingly, there is a significant variation between any given swimmer’s strengths and weaknesses across all strokes (McGibbon et al., 2018). Although swimmers might produce similar performance times, their pacing strategies within the splits might be drastically different (Saavedra et al., 2012; Knechtle et al., 2016).

To date, research showed that the analysis of pacing strategies in elite freestyle swimmers (Robertson et al., 2009; Knechtle et al., 2016; Lipinska et al., 2016). Further, there is limited data available on pacing strategies in 200 and 400 m individual medley in international swimming competitions in one study (Saavedra et al., 2012). However, pacing strategies in elite master level swimmers has yet to be examined. In the only study examining international swimmers in 200 and 400 m individual medley, it was observed that butterfly (1st split) was the fastest stroke regardless of final pacing and sex (Saavedra et al., 2012). When focusing on medalists in the 200 m event, it was observed that backstroke (3rd split) was the style that most correlated with performance whereas freestyle (front crawl, 4th split) correlated most strongly with the final race time in both men and women (Thompson et al., 2003). Interestingly, in the 400 m event, it was generally observed that breaststroke (3rd split) in men and freestyle in women were the styles most strongly correlated with the final time. However, when analyzed by groups, it was found that breaststroke was not the most important style for medalists. Considering medalists in Olympic Games, World Championships, European Championships, Commonwealth Games, Pan Pacific Games, U.S. Olympic Team Trials, and Australian Olympic Trials, backstroke was the style that most determined the final race time in the 200 and 400 m race in men, whereas it was backstroke (200 m) or freestyle (400 m) in women (Saavedra et al., 2012).

The present study aimed to investigate changes in swimming times by laps (i.e., splits in 50 m pools) in age group swimmers competing in the FINA WMC in 200 and 400 m individual medley. In elite individual medley swimmers, it was shown that the men applied a positive pacing strategy in the 200 and 400 m, whereas the women applied a less positive pacing strategy (Saavedra et al., 2012). The hypothesis was that male and female master swimmers would apply a similar pacing strategy like the elite male and female individual medley swimmers in international swimming competitions.

## Materials and Methods

### Ethical Approval

This study was approved by the Institutional Review Board of St. Gallen, Switzerland, with a waiver of the requirement for informed consent of the participants as the study involved the analysis of publicly available data.

### Procedures

To test our hypotheses, all female and male master swimmers competing in individual medley in 200 and 400 m in four FINA World Championships were included. All women and all men were included for every 5-year age groups starting from 25 years to ≥75 years to avoid a selection bias by analyzing only a limited sample of top athletes, such as the top 10 or top 100 of each age group. All data was sourced from the official and publicly accessible website of the FINA at www.fina.org/content/fina-masters-world-championships-results-archive. Results from 200 and 400 m individual medley were obtained from a total of four WMCs. To be eligible for FINA WMC,^1^ swimmers must be older than 25 years of age and must fulfil a qualification time (see for example www.fina.org/sites/default/files/general/2018-08-30_time_standards_-_gwangju.pdf) and be registered with an official swimming club. In the XV FINA WMC held in Montreal (Canada) in 2014, in the XVI FINA WMC held in Kazan (RUS) in 2015, in the FINA WMC held in Budapest (HUN) in 2017, and in the XVIII FINA WMC held in Gwangju (KOR) in 2019, for each distance, 200 and 400 m, and each swimmer, times for each 50 m length were recorded.

A total of 3,242 age group athletes (1,475 women and 1,767 men) who competed in 200 m (*n* = 2,300) and 400 m (*n* = 942) individual medley were considered. The age groups of 25–29 to 30–34, 35–39 to 40–44 years in 200 m races and 25–29 to 30–34, 30–34 to 35–39, 35–39 to 40–44, and 45–49 to 50–54 years in 400 m races were separated by women and men and 200 and 400 m.

### Statistical Analyses

All statistical procedures were carried out using the Statistical Package for the Social Sciences (SPSS version 26. IMB, Ill, USA) and GraphPad Prism (version 8.4.2. GraphPad Software LLC, CA, USA). The Shapiro-Wilk’s and Levene’s tests were applied for normality and homogeneity, respectively. Each participant had their mean and standard deviation (SD) calculated based on the partial race times (every 50 m) of the race. Individual mean and SD were further used to calculate an individual coefficient of variation (CV % formula: SD/mean*100) that was used a measure of pace variation. General linear models were as follows: race time (two-way ANOVA, separate for 200 and 400 m) sex × age group; pace variations (three-way ANOVA) sex × age group × distance; split/style race time (two-way ANOVA, separate for 200 and 400 m) sex × pace/style. Sex was always included as a fixed factor; all others were included as random factors. When interactions were found (*p* < 0.05), pairwise comparisons were applied to identify the differences more accurately. Partial eta squared (_p_*η*^2^) was used as estimates of effect size for the ANOVAs considering the following parameters partial eta-squared: small = 0.01; medium = 0.06; large = 0.14. The hypothesis of sphericity was verified by the Mauchly test and, when violated, the degrees of freedom are corrected by the Greenhouse-Geisser estimates. Significance level was set at 5% (*p* < 0.05).

## Results

**Figure 1:**
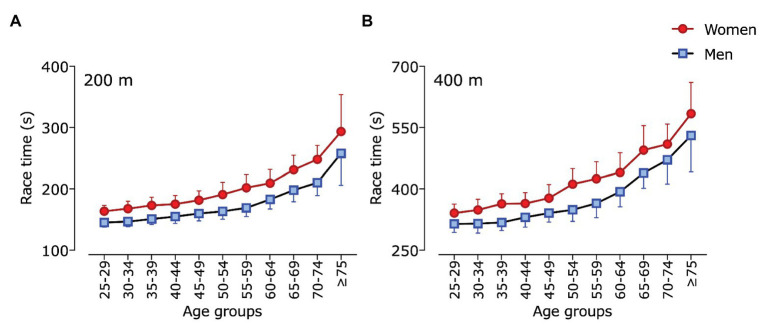
Mean race time for each age group in (A) 200 and (B) 400 m individual medley of men and women. Data are expressed as means and standard deviations (SDs).

The general linear model (two-way ANOVA) for race time in 200 and 400 m showed a significant effect for sex (200 m: *F* = 211.8; *p* < 0.001; _p_*η*^2^ = 0.952; 400 m: *F* = 140.7; *p* < 0.001; _p_*η*^2^ = 0.930), age group (200 m: *F* = 102.8; *p* < 0.001; _p_η^2^ = 0.990; 400 m: *F* = 113.1; *p* < 0.001; _p_*η*^2^ = 0.991), and interaction sex × age group (200 m: *F* = 5.1; *p* < 0.001; _p_*η*^2^ = 0.022; 400 m: *F* = 2.1; *p* < 0.001; _p_*η*^2^ = 0.022; Figure 1). Pairwise comparisons showed that men had lower (*p* < 0.001) race times than women among all age groups in both 200 and 400 m. Additionally, differences were found between almost all adjacent age groups, with the exception (*p* > 0.05) of age groups 25–29 to 30–34, 35–39 to 40–44 years in 200 m races; and 25–29 to 30–34, 30–34 to 35–39, 35–39 to 40–44, and 45–49 to 50–54 years in 400 m races.

**Figure 2:**
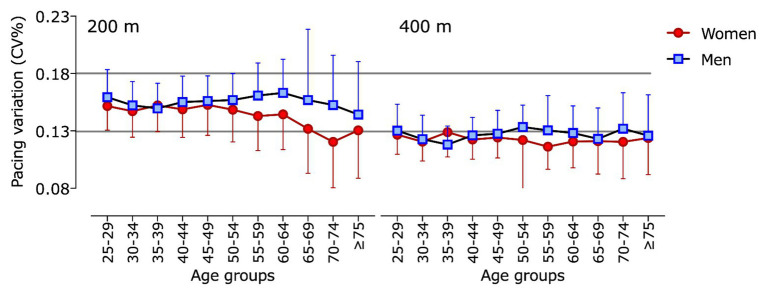
Mean pacing variation for each age group in 200 and 400 m individual medley of men and women. Data are expressed as means and SDs.

The general linear model (three-way ANOVA) for pace variation showed a significant effect for the interactions sex × age group (*F* = 3.8; *p* = 0.022; _p_*η*^2^ = 0.795) and sex × distance (*F* = 11.0; *p* = 0.006; _p_*η*^2^ = 0.491; Figure 2). Pairwise comparisons showed a higher pacing variation (*p* < 0.05) in 200 m races among all male age groups and all female age groups up to the age of 69 years old. Pace-variation pairwise comparisons between men and women showed no consistencies throughout age groups, with the exception of higher variation in men in age groups ≥55-year-old.

**Figure 3:**
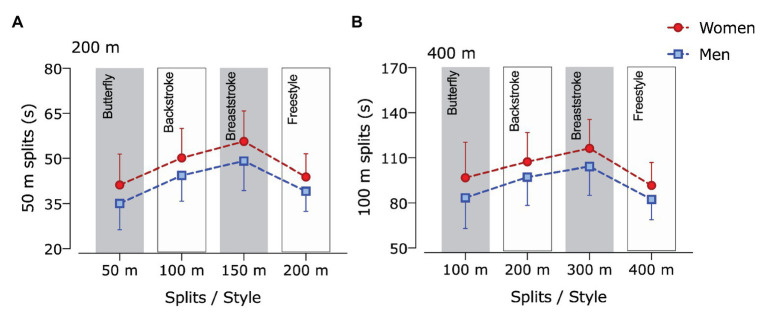
Mean race time in each split/style in (A) 200 and (B) 400 m individual medley of men and women. Data were expressed as means and SDs.

The last model with pacing/style as a factor showed a significant effect for sex (200 m: *F* = 256.7; *p* < 0.001; _p_*η*^2^ = 0.100; 400 m: *F* = 89.3; *p* < 0.001; _p_*η*^2^ = 0.087), for pacing/style (200 m: *F* = 10826.2; *p* < 0.001; _p_*η*^2^ = 0.825; 400 m: *F* = 11215.1; *p* < 0.001; _p_*η*^2^ = 0.923) and for an interaction sex × pace/style (200 m: *F* = 39.1; *p* < 0.001; _p_*η*^2^ = 0.017; 400 m: *F* = 22.5; *p* < 0.001; _p_*η*^2^ = 0.023; Figure 3). Pairwise comparisons showed that men were faster among all splits/style in both 200 and 400 m races, and significant changes were identified among each split/style for men and women in both 200 and 400 m races, being freestyle (4th split) the fastest, followed by butterfly (1st split), backstroke (2nd split), and breaststroke (3rd split).

## Discussion

In this study, we intended to investigate the pacing of master swimmers in individual medley for 200 and 400 m with the hypothesis that male master swimmers would apply a positive pacing strategy in the 200 and 400 m individual medley events, whereas female master swimmers would use a negative pacing strategy. Based on our results, the main findings were:

(i) freestyle was the fastest stroke, followed by butterfly, backstroke, and breaststroke, (ii) a higher pacing variation in the 200 m races compared to the 400 m races for all male age groups, and all female age groups up to 69 years old, and (iii) men were faster than women for all age groups in both the 200 and 400 m races.

A first important finding was that freestyle was the fastest stroke for both sexes followed by butterfly, backstroke, and breaststroke. This finding is in contrast to 200 and 400 m individual medley international swimming competitions, where butterfly was the fastest stroke for both sexes, and the least determinant stroke was breaststroke (Saavedra et al., 2012). Backstroke (200 and 400 m) correlated most with the final performance of men, whereas it was backstroke (200 m) and freestyle (400 m) in women (Saavedra et al., 2012). In lower classification, breaststroke was a determinant in the final performance in men, whereas it was backstroke for women (Moser et al., 2020). It might be that the competency in kinematic variables such as swimming speed, stroke rate and stroke length, and turns in freestyle stroke is higher and therefore, the age group swimmers were faster in freestyle stroke (Nikolaidis and Knechtle, 2017).

The present female and male master swimmers showed a final end spurt in both 200 and 400 m individual medley similar to female and male master freestyle swimmers competing in 200 and 400 m freestyle (Nikolaidis and Knechtle, 2017) but different to elite individual medley swimmers where elite male swimmers applied a positive pacing strategy in the 200 and 400 m individual medley events. In contrast, elite female swimmers used a negative pacing strategy (Saavedra et al., 2012). Potential explanations could be that elite swimmers adopt an all-out rather than pacing strategy (Mauger et al., 2012). Master swimmers could rely on their experience in pacing and start slow to finish fast (Nikolaidis and Knechtle, 2017), and/or master swimmers might rely on their ability for a fast end spurt compared to elite swimmers (Nikolaidis and Knechtle, 2017).

A second finding was a higher pacing variation in the 200 m races among all male age groups and all female age groups up to 69 years old. In this study, the CV increased in all males and females from the age group 65 years and above. Weir et al. (2002) noted that master level women dedicated more time to endurance and technique training than their male counterparts, who spent more time focusing on swimming speed and power training. However, younger age group swimmers could be more motivated with a stronger resilience and more stable psychological profile with a better pace control than older ones (Belinchón-deMiguel et al., 2019).

The last important finding was that men were faster than women across all age groups in 200 and 400 m. This finding confirms recent data in master butterfly swimmers where women were slower than men from 25 to 89 years (Knechtle et al., 2017) and also in master freestyle swimmers where men were faster than women for age groups 25–29 to 75–79 years but not for age groups 80–84 to 85–89 years (Knechtle et al., 2016). Previously, it has been demonstrated that swimming performance decreased progressively until approximately the age of 70 years, where the decrease becomes quadratic (Donato et al., 2003). Secondly, female swimmers experienced greater performance declines in sprint events but not in endurance performance as compared to their male counterparts (Donato et al., 2003). It has been shown that men have physiological advantages such as larger body size with more skeletal muscle mass, lower body fat, and a higher maximal delivery of anaerobic and aerobic energy (Sandbakk et al., 2018). This might be the reason why men had faster race times than women among all age groups in 200 and 400 m. However, increasing age seems to have no detrimental effect on swimming times in elderly athletes.

The present study is not free of limitations. It is important to note that an essential variable could be the environmental conditions like the air and water temperature in an outdoor pool (McMurray and Horvath, 1979; Saycell et al., 2019). A further limitation is that physiological variables were not available (Donato et al., 2003; Lazarus et al., 2019).

## Conclusion

In summary, men had lower race times than women among all age groups in 200 and 400 m. A higher pacing variation races in 200 m among all male age groups and all female age groups up to 69 years old. Men were faster among all splits/stroke in 200 and 400 m races. Freestyle (4th split) was the fastest stroke, followed by butterfly (1st split), backstroke (2nd split), and breaststroke (3rd split). The findings of the present study suggested that slower race times in both 200 and 400 m individual medley should be set as training goals for women compared to men for all age groups. In addition, when prescribing exercise for 400 m individual medley, strength and conditioning coaches should develop training programs corresponding to a relatively more even pacing. They may realize this aim by focusing on the slowest splits of 400 m individual medley (breaststroke and backstroke), i.e., a redistribution of the training load favoring these two strokes would be expected to decrease the variation of speed among strokes leading to a more even pacing.

## Data Availability Statement

The raw data supporting the conclusions of this article will be made available by the authors, without undue reservation.

## Author Contributions

All authors listed have made a substantial, direct and intellectual contribution to the work, and approved it for publication.

### Conflict of Interest

The authors declare that the research was conducted in the absence of any commercial or financial relationships that could be construed as a potential conflict of interest.

## Footnotes

^1^www.fina.org/discipline/masters
